# Identification of a novel transport system in *Borrelia burgdorferi* that links the inner and outer membranes

**DOI:** 10.1093/femspd/ftad014

**Published:** 2023-06-29

**Authors:** Hannah G Bowen, Melisha R Kenedy, David K Johnson, Alexander D MacKerell, Darrin R Akins

**Affiliations:** Department of Microbiology and Immunology, University of Oklahoma Health Sciences Center, 940 Stanton L. Young Blvd., BMSB 1053 Oklahoma City, OK 73104, United States; Department of Microbiology and Immunology, University of Oklahoma Health Sciences Center, 940 Stanton L. Young Blvd., BMSB 1053 Oklahoma City, OK 73104, United States; Shenkel Structural Biology Center, Molecular Graphics and Modeling Laboratory and the Computational Biology Core, University of Kansas, 2034 Becker Drive Lawrence, Kansas 66047, United States; Department of Pharmaceutical Sciences, School of Pharmacy, University of Maryland, Baltimore 20 North Pine Street Baltimore, Maryland 21201, United States; Department of Microbiology and Immunology, University of Oklahoma Health Sciences Center, 940 Stanton L. Young Blvd., BMSB 1053 Oklahoma City, OK 73104, United States

**Keywords:** lipopolysaccharide transport (LPT), glycolipids, lipoproteins, *Borrelia burgdorferi*, Site Identification by Ligand Competitive Saturation (SILCS)

## Abstract

*Borrelia burgdorferi*, the spirochete that causes Lyme disease, is a diderm organism that is similar to Gram-negative organisms in that it contains both an inner and outer membrane. Unlike typical Gram-negative organisms, however, *B. burgdorferi* lacks lipopolysaccharide (LPS). Using computational genome analyses and structural modeling, we identified a transport system containing six proteins in *B. burgdorferi* that are all orthologs to proteins found in the lipopolysaccharide transport (LPT) system that links the inner and outer membranes of Gram-negative organisms and is responsible for placing LPS on the surface of these organisms. While *B. burgdorferi* does not contain LPS, it does encode over 100 different surface-exposed lipoproteins and several major glycolipids, which like LPS are also highly amphiphilic molecules, though no system to transport these molecules to the borrelial surface is known. Accordingly, experiments supplemented by molecular modeling were undertaken to determine whether the orthologous LPT system identified in *B. burgdorferi* could transport lipoproteins and/or glycolipids to the borrelial outer membrane. Our combined observations strongly suggest that the LPT transport system does not transport lipoproteins to the surface. Molecular dynamic modeling, however, suggests that the borrelial LPT system could transport borrelial glycolipids to the outer membrane.

## Teaser

A novel transport pathway has been identified in the Lyme disease spirochete.

## Introduction

Lyme disease is a multisystem disorder that typically results in a combination of cardiac, neurological, dermatological, and rheumatological manifestations. Additionally, Lyme disease is a major public health problem as it is the most common tick-borne illness in both the USA and Europe (Schwartz et al. 2017, van den Wijngaard et al. 2017). *Borrelia burgdorferi* sensu stricto, the primary causative agent of Lyme disease in North America, can evade the host immune response for years without appropriate antibiotic treatment, which can result in a chronic and debilitating disease (Steere et al. 1987, Pachner 1988, Sehgal and Khurana 2015). *B. burgdorferi* is a diderm, extracellular pathogen, thus, the outer membrane (OM) is the interface between this spirochete and the infected host. Consequently, the OM has garnered much attention over the past several decades.

OM biogenesis studies in *B. burgdorferi* have unveiled that the spirochete has a quite unique OM as compared to Gram-negative organisms. Contrary to the very few surface-exposed lipoproteins in Gram-negative organisms, *B. burgdorferi* encodes over 100 lipoproteins located on its surface (Fraser et al. 1997, Dowdell et al. 2017). Depending on the environmental conditions, the lipoproteins are differentially expressed with only specific subsets being expressed at a given point in the organisms’ lifecycle (Ramamoorthy and Philipp 1998, Brooks et al. 2003, Ojaimi et al. 2003, Hyde et al. 2007, Angel et al. 2010). The *B. burgdorferi* surface lipoproteins are highly important to both immune evasion and overall disease pathogenesis (Brightbill et al. 1999, Hefty et al. 2001, Kenedy et al. 2012, Wilson and Bernstein 2016, Coburn et al. 2020). The *B. burgdorferi* OM also lacks the glycolipid lipopolysaccharide (LPS) (Takayama et al. 1987, Fraser et al. 1997), but does produce three other glycolipids (Hossain et al. 2001, Ben-Menachem et al. 2003, Stübs et al. 2011). These include cholesteryl 6-*O*-acyl-β-D-galactofuranoside (BbGL-I), mono-α-galactosyl-diacylglycerol (BbGL-II), and cholesteryl-β-D-galactopyranoside (Hossain et al. 2001, Ben-Menachem et al. 2003, Stübs et al. 2009, Szamosvári et al. 2022). Lyme disease patients develop antibodies specific to these glycolipids during infection indicating they are immunogenic during infection and could play an important role in disease pathogenesis (Schröder et al. 2008, Jones et al. 2009, Pozsgay et al. 2011). While the surface lipoproteins and glycolipids are crucial to the structure and function of the OM and are important with regard to disease pathogenesis, it is still unclear how these molecules are transported to the OM. It seems likely that the lipoprotein and glycolipid constituents of the OM would require the help of specific transport systems for proper localization.

Freeze-fracture electron microscopy has revealed that *B. burgdorferi* contains 10-fold fewer membrane-spanning OM proteins (OMPs) than *Escherichia coli* (Lugtenberg and van Alphen 1983, Radolf et al. 1994). Currently, only 10 different OMPs have been identified in *B. burgdorferi* (Sadziene et al. 1995, Skare et al. 1997, Parveen and Leong 2000, Cluss et al. 2004, Brooks et al. 2006, Antonara et al. 2007, Bunikis et al. 2008, Lenhart and Akins 2010, Kenedy et al. 2012, 2016, Wood et al. 2013, Shrestha et al. 2017). Among OMPs with known functions, only the OMP BamA has been determined to play a specific role in borrelial OM biogenesis as it is required for embedding other proteins into the OM (Lenhart and Akins 2010, Dunn et al. 2015, Iqbal et al. 2016). This is analogous to the role of BamA and the greater beta-barrel assembly machinery (BAM) complex in Gram-negative organisms (Kim et al. 2012). Another critical system in Gram-negative OM biogenesis is the lipopolysaccharide transport (LPT) system. The LPT system transports LPS to the surface of Gram-negative organisms. During this process, LPS is extracted from the periplasmic leaflet of the inner membrane and transported through the periplasm before being inserted into the outer leaflet of the OM (Sperandeo et al. 2008, Freinkman et al. 2012, Laguri et al. 2017). The LPT complex contains seven different proteins, and they are essential for LPS surface localization in Gram-negative organisms (Sampson et al. 1989, Sperandeo et al. 2006, Wu et al. 2006, Ruiz et al. 2008). The LPT system contains an inner membrane permease unit comprised of an ATP-binding cassette homodimer of LptB and a permease made of LptF and LptG. Together, these units extract LPS from the inner membrane and transport LPS to the LptC protein, which is found in the periplasm but is anchored to the inner membrane by a single transmembrane domain (Simpson et al. 2016). The periplasmic bridge protein LptA interacts with LptC and accepts LPS before transporting LPS to the OMP LptD, which is the terminal component of the transport system and is required to localize LPS into the outer leaflet of the OM (Bowyer et al. 2011, Sperandeo et al. 2011, Dong et al. 2017a, Hicks and Jia 2018). Additionally, in Gram-negative organisms there is an LptE protein that interacts with LptD that helps to terminate LPS transport (Wu et al. 2006, Botte et al. 2022). LptE plays several important roles: it is necessary for proper folding of the OMP LptD, it prevents aggregation of LPS during the transport and release process, and it also acts as a plug for the beta-barrel pore formed by LptD, which prevents continual and unabated LPS transport (Ruiz et al. 2010, Freinkman et al. 2011, Chng et al. 2012, Malojčić et al. 2014). A unique feature of the LPT system is the presence of a beta-taco fold (commonly incorrectly described as a beta-jelly roll fold) in the LPT proteins LptF, LptG, LptC, LptA, and LptD, and this unique structure allows for the transport of amphiphilic molecules such as LPS (Hicks and Jia 2018).

*B. burgdorferi* has previously been reported to contain orthologs for five out of the seven LPT proteins (Putker et al. 2015). We have also previously reported that *B. burgdorferi* protein BB0838, encoded by open reading frame (ORF) *bb0838*, is a surface exposed OMP with a computationally predicted structure similar to that of LptD (Kenedy et al. 2016). Pukter et al. (2015) identified BB0465, BB0466, BB0807/BB0808, and BB0838 as orthologs to LptA, LptB, LptF/LptG, and LptD, respectively. Here, we describe the identification of *B. burgdorferi* protein BB0464, an ortholog to LptC, and provide evidence that BB0807 is the LptF ortholog and that BB0808 is the LptG ortholog. Finally, we have also generated a working model of the LPT system in *B. burgdorferi* (consisting of proteins encoded by ORFs *bb0464, bb0465, bb0466, bb0807, bb0808*, and *bb0838*) and empirically determined that these proteins interact as would be expected of an LPT system. While the presence of a putative LPS transport system in an organism that lacks LPS is counterintuitive, we should note that *B. burgdorferi* does contain an abundance of surface-exposed lipoproteins and multiple glycolipids that are similar in their amphiphilic nature to LPS. Our combined data indicate that the novel borrelial LPT system does not transport lipoproteins to the surface and suggest that the borrelial glycolipids are the cargo instead.

## Methods

### Bacterial strains and growth conditions

*B. burgdorferi* strain B31 was cultivated at 34°C in BSK-II liquid medium containing 6% heat-inactivated rabbit serum (BSK-II complete, pH 7.6). *B. burgdorferi* strain B31-5A4 LK (Gilbert et al. 2007) was cultivated at 34°C BSK-II complete containing 200 ug/ml kanamycin, and *B. burgdorferi* strain B31-5A4 LK-*flacp::bblptD* was cultivated at 34°C in BSK-II complete with 200 ug/ml kanamycin, 100 ug/ml streptomycin, and either 0, 0.01, or 1 mM isopropyl-β-d-thiogalactopyranoside (IPTG).

All pACYCDuet-1 (EMD Millipore, Burlington, MA) cloning vectors were propagated in *E. coli* strain DH5α for plasmid construction and transformed into *E. coli* Overexpress™ C41(DE3) (Lucigen Corp, Middleton, WI) for expression. *E. coli* was grown at 37°C in Luria–Bertani (LB) broth or on LB agar supplemented with chloramphenicol.

### Recombinant protein and antibody production

Utilizing oligonucleotides BbLptD F and BbLptD R (Table 1), the N-terminal domain of BbLptD was amplified from nucleotide 88 to nucleotide 873. The amplicon was digested with restriction enzymes NheI and XhoI and ligated into an NheI/XhoI digested pET23a (EMD Millipore, Billerica, MA). The resulting construct was transformed into *E. coli* Overexpress™ C41(DE3) for expression. The recombinant protein was purified using nickel-nitrilotriacetic acid agarose (Ni-NTA; Qiagen, Valencia, CA) in His-tag native and His-tag denaturing purification conditions as described previously (Luthra et al. 2011, Kenedy et al. 2014). A 70% native and 30% denatured mix of the recombinant protein was used for antibody generation. Rat polyclonal antibodies specific for the N-terminal domain of BbLptD (amino acids 29–291) were generated by Envigo (Indianapolis, IN). Rat polyclonal antibodies specific to OspA, OspC, CspA, or P66, and rabbit polyclonal antibodies specific to FlaB were generated as previously described (Radolf et al. 1994, Cox et al. 1996, Brooks et al. 2005, 2006, Kenedy et al. 2014). These antibodies were subsequently utilized for immunofluorescence assays and immunoblots.

**Table 1.** Oligonucleotides used in this study.

Primer name	Sequence (5′–3′, restriction sites in bold)	Description
BbLptD F	GCG**GCTAGC**CAGACTATAGATGATGAAAATTC	Antibody production (nucleotides 88–873)
BbLptD R	GCG**CTCGAG**ACTATCTCCCGGTCTGAAAAAA	
BbLptDNT-S F	GCG**CATATG**CAGACTATAGATGATGAAAATTC	Cloning the N-terminal domain of *bblptD* into S tag of pACYCDuet-1 (nucleotides 90–813)
BbLptDNT-S R	GCG**GGTACC**TATGGCATTTAAAAACCCAAAATC	
BbLptA-S F	GCG**CATATG**ACACAAATAGAATCCAGCCTTA	Cloning *bblptA* into S tag of pACYCDuet-1 (nucleotides 60–end)
BbLptA-S R	GCG**GGTACC**TTTTTTTTCTTCAGAAGCATCATT	
BbLptA-His F	GCG**GGATCC**TACACAAATAGAATCCAGCCTTA	Cloning *bblptA* into 6xHis tag of pACYCDuet-1 (nucleotides 60–end)
BbLptA-His R	GCG**GAGCTC**TTATTTTTTTTCTTCAGAAGCATC	
BbLptC-His F	GCG**GGATCC**TTCTAGTAGATCTGATGTGGC	Cloning *bblptC* into 6xHis tag of pACYCDuet-1 (nucleotides 69–end)
BbLptC-His R	GCG**GAGCTC**TCAATTCATGATTCCTTCAACTC	
OspC-S F	GCG**CATATG**GGGAAAGATGGGAATACATCT	Cloning *ospC* into S tag of pACYCDuet-1 (nucleotides 69–end)
OspC-S R	GCG**GGTACC**AGGTTTTTTTGGACTTTCTGC	
BbLptD up mutant F	GCG**GGTACC**ACAGAAATTAGGGAGCTTTTTG	Cloning 600 base pairs upstream of *bblptD*
BbLptD up mutant R	GCG**CTCGAG**CTATAAAAGATTTTTTAAAAACATTCC	Cloning 600 base pairs upstream of *bblptD*
BbLptD down mutant F	GCG**GGATCC**CATATGCGAGAATTCCTATACAGGAATG	Cloning 600 base pairs downstream of *bblptD* start
BbLptDdown mutant R	GCG**TCTAGA**GGTAACATCATTGTCTATCTTTT	Cloning 600 base pairs downstream of *bblptD* start

### *B. burgdorferi* LPT-orthologous system identification and protein modeling

BbLptD was first identified as a putative ortholog to LptD in a previous publication (Kenedy et al. 2016). The National Center for Biotechnology Information BLAST analysis (https://blast.ncbi.nlm.nih.gov/Blast.cgi) was performed using *E. coli* proteins LptA, LptC, LptB, LptF, and LptG, limiting results to those that belong to the genus *Borrelia*. Subsequent computational modeling was performed on the following *B. burgdorferi* Lpt orthologs: BB0838 (LptD), BB0465 (LptA), BB0464 (LptC), BB0466 (LptB), BB0807 (LptF), and BB0808 (LptG). The proteins were modeled with AlphaFold2.1 (Jumper et al. 2021, Varadi et al. 2021) with the default settings, and the top model was chosen by pLDDT. Both BbLptA and BbLptD were modeled without their predicted signal peptide sequence, as predicted by PrediSi, Signal-CF, and Signal-P.

The working model of the *B. burgdorferi* LPT-orthologous system was created utilizing the models of individual proteins obtained from AlphaFold2. BbLptD and BbLptA were inserted into the model as individual proteins. BbLptC, BbLptF, and BbLptG were inserted into the model as a trimer. BbLptB was inserted as a homodimer. The working model was created using BioRender (https://biorender.com/).

Multimer modeling was performed using AlphaFold2.1 (Evans et al. 2022). For each multimer, the proteins of interest were submitted together to determine the likelihood of interaction and at what interface. BbLptA (residues 20–231) and BbLptDNT (residues 30–271) were modeled together, both in the absence of their respective signal peptides. BbLptA (residues 20–231) was modeled with BbLptC (residues 19–174), both without their signal peptides. BbLptC was submitted for multimer modeling with BbLptF and BbLptG separately, followed by BbLptC, BbLptF, and BbLptG together. For these modeling predictions, the full-length proteins were submitted. Of the 25 models generated, one preferred model was selected by visual inspection, ipTM + pTM, and pLDDT scores at the modeled interface.

### Modeling of glycolipid cargo in BbLptA

Modeling of BbGL-I and BbGL-II inside BbLptA was performed using a combination of Site Identification by Ligand Competitive Saturation (SILCS), Rapid Overlay of Chemical Structures (ROCS) by OpenEye, and MD using GROMACS (Guvench and MacKerell 2009, ROCS 3.5.1.1 OpenEye 2007, OpenEye Scientific Software 2010, Hawkins et al. 2007, 2010, Abraham et al. 2015). SILCS simulations were performed on the model of BbLptA, generating 3D maps of functional group affinities patterns (FragMaps), as well as an exclusion map where functional groups or water are forbidden from interacting. Identification of possible binding sites for BbGL-I was performed by decomposing the molecule into smaller fragments that were used with SILCS-Hotspots to identify all sites with interaction energies stronger than −2 kcal/mol. Within the beta-taco fold of BbLptA, there was a hotspot for the sterol of BbGL-I, two hotspots for octane, and two hotspots for 2-methylbutane. The coordinates for the lowest energy conformation of each probe at its respective hotspot(s) were used as a pharmacophore to place BbGL-I within the beta-taco. A total of 27 551 unique, low-energy conformations of BbGL-I were generated using OMEGA and overlaid on the pharmacophore based on optimization of shape/chemical complementarity using ROCS, both developed by OpenEye (ROCS 3.5.1.1 OpenEye 2007, OpenEye Scientific Software 2010, Hawkins et al. 2007, 2010). The top 10 scoring conformations were evaluated, and one pose was selected based on maximal overlap with minimal clashes with the protein backbone. The conformer was superimposed into the beta-taco. MD was performed using GROMACS with the CHARMM36 m forcefield (Abraham et al. 2015, Huang et al. 2017). The ligand was parameterized using CGenFF (Vanommeslaeghe et al. 2010), and the protein–ligand complex was solvated in a dodecahedron unit cell with a sodium added to neutralize the system. A steepest decent minimization was performed, and the system was equilibrated at 300 K with a 100-ps NVT simulation followed by an additional 100 ps NPT simulation. Finally, a 200-ns NPT production simulation was performed. BbGL-II was modeled using a similar protocol. A total of 26 635 conformers were generated using Omega and overlaid to the final BbGL-I pose from the MD simulation. MD was performed using the aforementioned protocol.

### Localization immunofluorescence assays

For experiments utilizing *B. burgdorferi* strain B31, cells were grown to mid-exponential phase and diluted to 5 × 10^6^ organisms/ml. Cell suspensions were coincubated for 1 h with rat-anti-BbLptDNT antibodies at a dilution of 1:100 and rabbit-anti-FlaB antibodies at a dilution of 1:2500. As a control, separate cell suspensions were coincubated for 1 h with rat-anti-CspA antibodies at a dilution of 1:100 and rabbit-anti-FlaB antibodies at a dilution of 1:2500. Cells were washed three times with 1X PBS, and the final pellet was resuspended in 100 μl of 1X PBS. A volume of 10 μl of the final resuspension were spotted onto a microscope slide, dried overnight, and fixed with acetone. Samples were blocked for 30 min with PBS containing 0.2% bovine serum albumin (BSA). Fixed and blocked samples were then incubated for 45 min with Alexa Fluor 488-conjugated goat-anti-rat antibodies (Invitrogen, Waltham, MA) at a dilution of 1:250 and Alexa Fluor 568-conjucated goat-anti-rabbit antibodies (Invitrogen) at a dilution of 1:1000. Samples were washed three times with PBS containing 0.2% BSA, mounted with one drop of a 1:1 mixture of Vectashield mounting solution containing 4’,6-diamidino-phenylindole (DAPI; Vector Laboratories, Burlingame, CA) and Vectashield mounting solution without DAPI (Vector Laboratories), and sealed with a coverslip. Another set of cell suspensions were incubated without any antibodies, washed, resuspended, spotted, and fixed. The fixed cells were then blocked with PBS containing 0.2% BSA, incubated with the same dilutions and mixtures of primary antibodies for 45 min. The previous description of the protocol postprimary antibody was then performed the same.

Experiments utilizing *B. burgdorferi* strain B31-5A4 LK and B31-5A4 LK-*flacp::bblptD* were performed identically with the exception of antibodies and *B. burgdorferi* strains utilized. These strains were coincubated with a combination of rat-anti-CspA antibodies at a dilution 1:100 and rabbit-anti-FlaB antibodies at a dilution of 1:2500 for 1 h at room temperature. The remaining experimental procedure was performed exactly as described above. Images of samples were visualized and captured with an Olympus BX-60 fluorescence microscope and Olympus DP27 camera (Olympus America Inc., Center Valley, PA).

### Cloning of *B. burgdorferi* Lpt-orthologs for copurification assays

The DNA sequences of all putative Lpt-orthologs were amplified from *B. burgdorferi* B31 genomic DNA using the primers in Table 1. Amplicons were cloned into one of two multiple cloning sites of pACYCDuet-1, depending on the desired tag. Amplicons destined for the 6x His tag were digested and cloned into the BamHI and SacI sites of pACYCDuet-1. Amplicons destined for the S tag were digested and cloned into the NdeI and KpnI sites of pACYCDuet-1. Cloning into each multiple cloning site was performed stepwise with complete insertion into the 6x His-tag and then complete insertion into the S-tag. Vectors were transformed into *E. coli* Overexpress™ C41(DE3) for protein expression. Sequences of inserts were verified through DNA sequencing to ensure the genes of interest were unaltered during the cloning process.

### His-tag native purification for copurification assays

For the copurification assays, pACYCDuet constructs in *E. coli* stain C41 were grown overnight at 37°C in 35 ml LB cultures. The 35 ml of starter culture was inoculated into 500 ml of fresh LB and grown at 37°C to an optical density at 600 nm (OD_600_) of between 0.55 and 0.75. At this point, protein expression was induced with 1 mM IPTG followed by an additional incubation for 3 h at 37°C. Cells were pelleted at 8200 × *g* for 20 min at 4°C. Cell pellets were resuspended in 15 ml of lysis buffer (50 mM NaH_2_PO_4_, 300 mM NaCl, 10 mM imidazole, pH 8) with 15 μl of protease inhibitor cocktail. Resuspended cells were lysed by sonication and pelleted at 12 500 × *g* for 45 min. The resulting supernatant was normalized to an OD_600_ of 0.044 and 15 ml of the supernatant was incubated with 2.5 ml bed volume of Ni-NTA agarose for 15 min to bind the His-tagged proteins to the agarose. The resin was washed with 100 ml of wash buffer (50 mM NaH_2_PO_4_, 300 mM NaCl, 20 mM imidazole, pH 8), followed by 50 ml of increased imidazole concentration wash buffer (50 mM NaH_2_PO_4_, 300 mM NaCl, 40 mM imidazole, pH 8). Proteins were not eluted, as solubility of the proteins drops drastically upon elution.

### SDS-PAGE and immunoblot analysis for copurification assays

Supernatant samples were prepared by mixing 1:1 with final sample buffer [FSB; 62.5 μM Tris-HCl (pH 6.8), 10% (v/v) glycerol, 5% (v/v) β-mercaptoethanol, 5% SDS, 0.001% bromophenol blue] and boiled for 10 min. Copurification samples were prepared by taking 200 μl of slurry of each final sample bound to Ni-NTA agarose, removing the wash buffer, resuspending in 160 μl of FSB, and boiling for 10 min. Samples were run by electrophoresis on SDS-PAGE gels with 2.4% stacking and 12.5% separating. For the anti-His immunoblots, every normalized supernatant was loaded at 6 ul of the 1:1 sample and 5 μl of every copurification resin sample was loaded. This was loaded the same for the anti-S immunoblots with the exception of the supernatant samples containing OspC-S. This protein is expressed at significantly higher levels than all other proteins in the experiment, so to obtain images, the supernatant samples containing this protein for the anti-S immunoblots was diluted 384x. Copurification resin samples, however, were loaded the same for OspC-S containing samples as all other samples. Gels were transferred electrophoretically to PVDF membrane (polyinylidene fluoride; BioRad, Hercules, CA) for immunoblot analysis. All immunoblots were performed as previously described (Iqbal et al. 2016). To analyze the presence of S-tagged recombinant proteins, immunoblots were incubated with 1:2000 mouse-anti-S antibody (EMD Millipore) followed by a 45-min room temperature incubation with horseradish peroxidase-conjugated goat-anti-mouse secondary antibody (BioRad). To analyze the presence of His-tagged recombinant proteins, immunoblots were incubated with 1:2000 HRP-conjugated mouse-anti-His antibody (R&D Systems, Minneapolis, MN) for 1.5 h. Immunoblots were developed with enhanced chemiluminescence (ECL) substrate (Thermo Fisher Scientific, Waltham, MA) and subsequently visualized with the ChemiDoc MP Imaging System (BioRad). For a loading control, 6 μl of all OD_600_ normalized supernatants were immunoblotted with 1:4000 mouse-anti-GAPDH antibody (Invitrogen), followed by 1:4000 horseradish peroxidase-conjugated goat-anti-mouse secondary antibody (BioRad).

### Generation of *B. burgdorferi* strain B31-5A4 LK-*flacp::bblptD*

The BbLptD IPTG regulatable mutant, designated *flacp::bblptD*, was generated by inserting the *flacp* promoter immediately upstream of the start codon of *bblptD* as previously described (Iqbal et al. 2016). The primers used to make the construct are listed in Table 1 with the restriction enzymes used for cloning into the pBluescript-II SK + vector (Stratagene, La Jolla, CA). Primers “ BbLptD up mutant F” and “ BbLptD up mutant R” were utilized to amplify 600 bp upstream of the start codon of *bblptD* and primers “ BbLptD down mutant F” and “ BbLptD down mutant R” were utilized to amplify 600 bp downstream of the start codon of *bblptD*. The amplicons were inserted into the multiple cloning site of pBluescript-II SK + vector using the corresponding restriction enzymes/sites. The streptomycin resistance cassette and the *flacp* promoter were digested as a unit from the pTLflacp::795 construct that was described previously (Lenhart and Akins 2010) and inserted into the XhoI and NdeI sites of the construct. The subsequent construct was electroporated into *B. burgdorferi* B31-5A4 LK and grown in BSK-II media supplemented with 1 mM IPTG, kanamycin, and streptomycin as described above. Clones were screened by PCR to verify presence of all *B. burgdorferi* plasmids and by immunoblot to confirm IPTG-regulation of BbLptD.

### Proteinase K (PK) accessibility assays

Proteinase K experiments were performed as previously described (Brooks et al. 2005) with *B. burgdorferi* strains B31-5A4 LK or B31-5A4 LK-*flacp::bblptD* supplemented with 0, 0.01, or 1 mM IPTG. Final samples were resuspended in FSB and boiled for 10 min. For immunoblot analysis, samples were subjected to SDS-PAGE electrophoresis and transferred electrophoretically to PVDF membrane. These samples were immunoblotted with rat-anti-CspA or rat-anti-P66 antibodies, both at a dilution of 1:4000, followed by incubation with horseradish peroxidase-conjugated goat-anti-rat antibodies at a dilution of 1:8000. For FlaB immunoblots, samples were incubated with rabbit-anti-FlaB antibody at a dilution of 1:15 000, followed by secondary incubation with horseradish peroxidase-conjugated goat-anti-rabbit antibodies at a dilution of 1:30 000. All immunoblots were developed and visualized as described above.

## Results

### Genetic organization of the orthologs that comprise the *B. burgdorferi* LPT system

**Figure 1.**
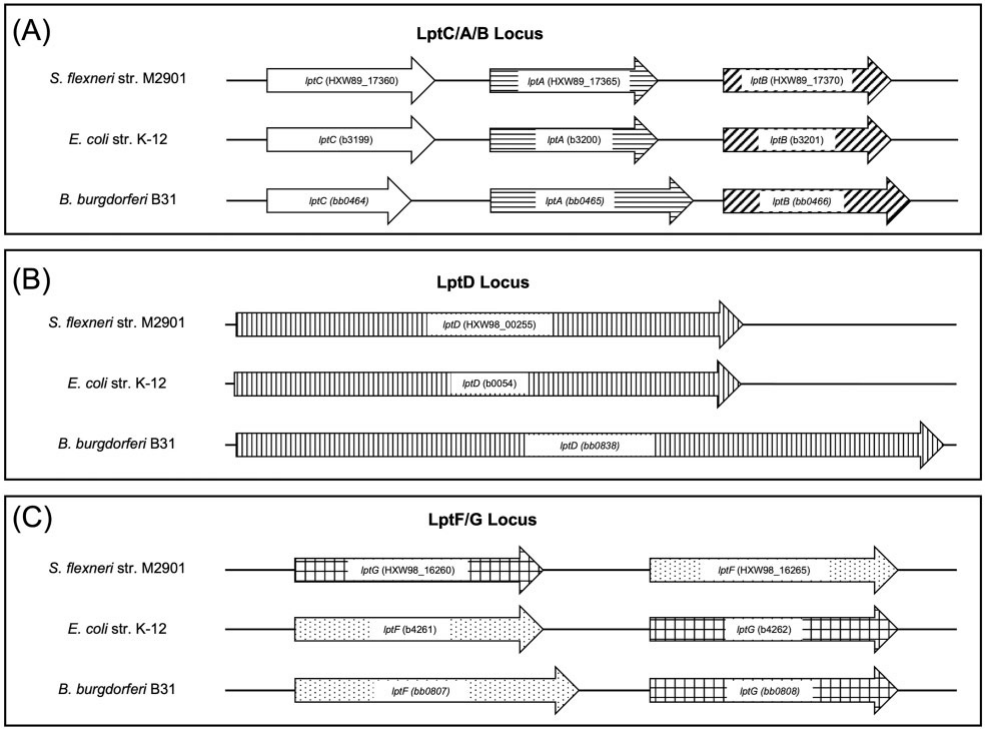
Comparison of the genetic organization of the LPT systems of *B. burgdorferi* strain B31, *S. flexneri* strain M2901, and *E. coli* strain K-12. Lpt genes are encoded at three distinct loci in *B. burgdorferi*, which is similar to the genetic organization found in *E. coli* and *S. flexneri*. (A) Unfilled arrows indicate genes encoding LptC proteins. Horizontal line-filled arrows indicate genes encoding LptA proteins. Diagonal line-filled arrows indicate genes encoding LptB proteins. (B) Vertical line-filled arrows indicate genes encoding LptD proteins. (C) Dot-filled arrows indicate genes encoding LptF proteins. Checkered-filled arrows indicate genes encoding LptG proteins. The length of arrows represents the respective size of the gene compared to its ortholog.

The predicted *B. burgdorferi* LPT transport system is comprised of six proteins. We have previously identified *B. burgdorferi* protein BB0838 as a 120-kDa, membrane-spanning OMP and determined that it is an ortholog to LptD (Kenedy et al. 2016). Previous studies have also identified BB0838 as an LptD ortholog and proposed additional borrelial ORFs *bb0465, bb0466*, and *bb0807*/*bb0808* as *lptA, lptB*, and *lptF/G* orthologs, respectively (Putker et al. 2015). Scanning the genome and additional computational modeling allowed us to identify the protein BB0464 as a predicted LptC ortholog. BLASTP analyses (blast.ncbi.nlm.nih.gov/Blast.cgi) identified the respective Lpt orthologs as the top query hits for the *B. burgdorferi* protein sequences, further confirming the orthology between the respective *B. burgdorferi* proteins and the Lpt proteins. Consistent with these computer-based analyses, the overall genomic organization of the genes encoding the borrelial orthologs was found to be identical to the organization of the LPT genes from the enteric pathogens *Shigella flexneri* and *E. coli* (Fig. 1). Specifically, these proteins are encoded at three distinct loci on the chromosome of each bacterium. Proteins LptC, LptA, and LptB are encoded in *S. flexneri* and *E. coli* at one locus, and the respective orthologs from *B. burgdorferi*, ORFs *lptC* (*bb0464*), *lptA* (*bb0465*), and *lptB* (*bb0466*), are also encoded at one locus in the same order (Fig. 1A). LptD is encoded at a second distinct locus in *S. flexneri, E. coli*, and in *B. burgdorferi* (Fig. 1B). Finally, the inner membrane permease units, LptF and LptG, are encoded in *S. flexneri* and *E. coli* at a third distinct locus and the same organization is found in *B. burgdorferi* (Fig. 1C). We have denoted BB0807 as the LptF ortholog and BB0808 as the LptG ortholog, which is most consistent with better computational alignment scores for each pair using PyMOLv2.4.0 (Schrödinger 2020) (data not shown).

### Structural models of *B. burgdorferi* Lpt orthologs

**Figure 2.**
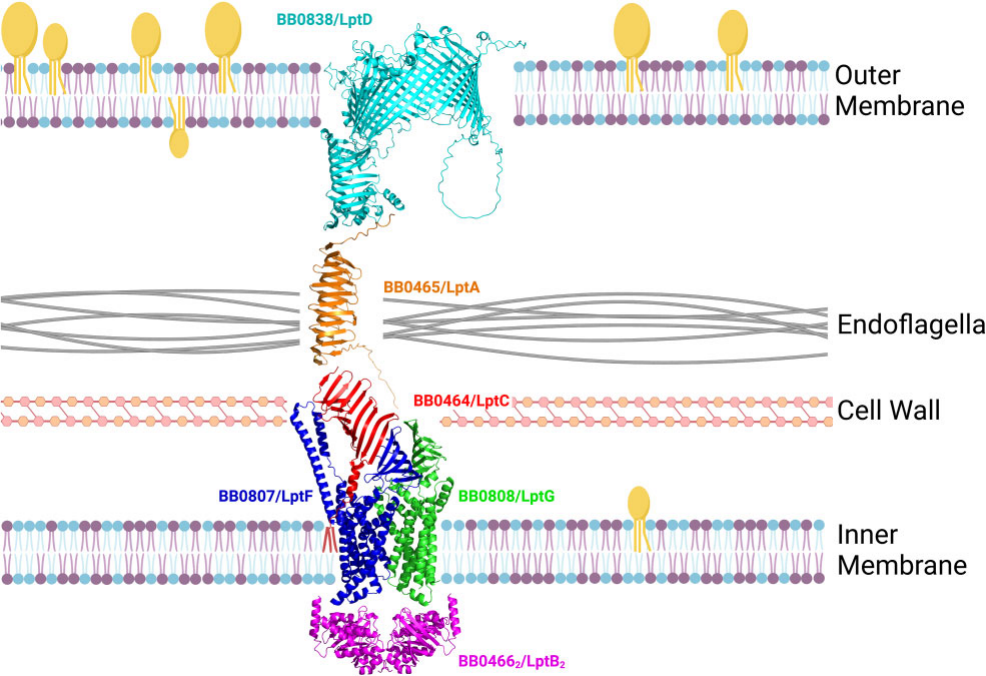
Current model of the *B. burgdorferi* LPT-orthologous system. The *B. burgdorferi* LPT system deduced from the combined genetic organization and AlphaFold2.1 structural modeling of each *B. burgdorferi* Lpt ortholog. BB0838 is shown in cyan, BB0465 is shown in orange, BB0464 is shown in red, BB0807 is shown in blue, BB0808 is shown in green, and BB0466_2_ is shown in magenta. These proteins are colisted with their predicted Lpt ortholog. Fatty acid tails of the putative lipoprotein BB0464 are shown in red. Tri-acylated, yellow, globular molecules in the membranes represent borrelial lipoproteins. Di-acylated blue molecules in the membrane represent borrelial phospholipids. Di-acylated purple molecules represent borrelial glycolipids. This figure was created with BioRender.

Through structural modeling of each Lpt ortholog in *B. burgdorferi*, we have predicted a working model for the *B. burgdorferi* LPT system (Fig. 2), that is similar to what has been described for the LPT system in Gram-negative organisms. Taking advantage of advances in computational 3D protein structure prediction, we utilized the AlphaFold2.1 modeling algorithm (Jumper et al. 2021, Varadi et al. 2021) to generate individual models of the *B. burgdorferi* Lpt-orthologs (Fig. 2). The LptD ortholog, BB0838, is predicted to fold into a large, OM-spanning beta barrel beginning approximately at residue 294 and continuing through the C-terminus of the amino acid sequence (Fig. 2). The beta barrel portion of BB0838 is predicted to be larger than the *E. coli* LptD and contain 31 beta strands rather than 26. BB0838 also was predicted to include a large, periplasmic loop between beta strands 10 and 11 (Fig. 2). Additionally, BB0838 contains a predicted periplasmic, N-terminal domain comprised of residues 30–293 (Fig. 2). Residues 76–293 take the conformation of a beta-taco fold motif (Fig. 2). The N-terminal beta-taco fold motif has also been identified and characterized in the solved crystal structure of *E. coli* LptD (Botos et al. 2016). Interestingly, residues 30–75 of the BB0838 N-terminal domain consist of a series of four alpha helices, that are not present in other LptD proteins (Botos et al. 2016). The AlphaFold2.1 algorithm provides a predicted local distance difference test (pLDDT) as a per-residue confidence score for each model. Accordingly, an average pLDDT ≥ 90 indicates very high confidence in the model, a pLDDT from 70 to 89.99 is considered confident, a pLDDT from 50 to 69.99 is considered low confidence, and any pLDDT score lower than 50 corresponds to very low confidence (Jumper et al. 2021, Varadi et al. 2021). As shown in Table 2, the predicted BB0838 model resulted in a pLDDT of 72.70, indicating a confident score for the model shown in Fig. 2. Interestingly, the confidence in the N-terminal domain of BB0838 modeled alone (86.40) is higher than that of the beta barrel of BB0838 modeled alone (72.10) or BB0838 as a whole (Table 2). This was not surprising given the highly specialized role the N-terminal beta-taco fold of LptD has in the transport of LPS (Sperandeo et al. 2008, Gu et al. 2015, Laguri et al. 2017). With this data, we conclude that BB0838 is indeed the *B. burgdorferi* ortholog of LptD.

**Table 2.** AlphaFold2.1 pLDDT scores of *B. burgdorferi* Lpt orthologs.

Protein	pLDDT^†^
BB0464	90.78
BB0465	87.96
BB0466	92.16
BB0807	84.71
BB0808	81.80
BB0838	72.70
BB0838NT^‡^	86.40
BB0838BB^§^	72.10

†: The pLDDT per-residue score is averaged to give the numbers shown for each respective protein.

‡: BB0838NT indicates the N-terminal domain (residues Q30–I271) was modeled alone.

§: BB0838BB indicates the transmembrane beta-barrel (residues F272–K1146) was modeled alone.

*B. burgdorferi* BB0465 (residues 20–231; signal peptide excluded) is predicted to fold into a beta-taco formation similar to the N-terminal domain of BB0838 (Fig. 2). When modeled with the AlphaFold2.1 algorithm, the predicted structure of BB0465 has a confident pLDDT score of 87.96 (Table 2). Further, this model is very similar to that of the known crystal structure of *E. coli* LptA, which contains the same highly specialized beta-taco fold structure as predicted for BB0465 (Suits et al. 2008). These findings combined with the genomic and BLASTP data allow us to confirm that BB0465 is the periplasmic bridge ortholog, LptA, in the *B. burgdorferi* LPT system. As shown in Fig. 2, the AlphaFold2.1 model of BB0464 resembles the known crystal structure of LptC, which is characterized by a periplasmic beta-taco fold with a single alpha helix anchoring LptC into the inner membrane (Tran et al. 2010). Contrary to other LptC proteins, however, the first 14 residues of BB0464 are predicted as a signal II peptide with a cysteine residue directly after the predicted cleavage site. Because of this, it seems likely that BB0464 is an inner membrane lipoprotein as suggested by He et al. (2023) and is illustrated as so in Fig. 2. This would indicate the mechanism of inner membrane anchoring of the *B. burgdorferi* LptC ortholog diverges from canonical LptC membrane anchoring. Overall, the model of BB0464 is of very high confidence with a pLDDT of 90.78 (Table 2), leading us to predict that BB0464 is the LptC ortholog.

*B. burgdorferi* BB0807, BB0808, and BB0466 were initially modeled with AlphaFold2.1 separately to obtain their individual pLDDT scores (Table 2). The predicted structure of BB0466 (Fig. 2) is very similar to the known structure of various Gram-negative LptB homodimers (Owens et al. 2019) and contains motifs classified in the ATP-binding cassette protein family. Combined with the very high pLDDT of 92.16 (Table 2), we conclude that BB0466 is the predicted LptB ortholog. The predicted structural models of BB0807 and BB0808 shown in Fig. 2 are also considered confident with respective pLDDT scores of 84.71 and 81.80 (Table 2). While generally, LptF and LptG are similar in their structure (Dong et al. 2017b, Owens et al. 2019, Tang et al. 2019), we wanted to determine which of the *B. burgdorferi* putative orthologs was most similar to either LptF or LptG. To do this, we utilized the solved crystal structure of the *Enterobacter cloacae* LptB_2_FGC complex (PDB 6MIT) (Owens et al. 2019). By aligning BB0807 and BB0808 individually to the LptF and LptG of this model in PyMOLv2.4.0, we were able to determine that the root-mean-square deviation (RMSD) was lowest when BB0807 was aligned with LptF and BB0808 was aligned with LptG (data not shown). The lower RMSD score indicates a higher confidence level that BB0807 is the LptF ortholog and that BB0808 is the LptG ortholog. BB0807 and BB0808 are predicted to have the same small beta-taco fold domain and larger inner membrane, transmembrane domain comprised of several alpha helices similar to what has been observed in Gram-negative LptFG heterodimers (Owens et al. 2019). To create a more cohesive and interaction-representative model, BB0807, BB0808, and BB0464 were also modeled together as a trimer using the AlphaFold2.1 algorithm, which is what is illustrated in the working model (Fig. 2). From this point forward for more clarity, the *B. burgdorferi* Lpt orthologs BB0464, BB0465, BB0466, BB0807, BB0808, and BB0838 will be referred to as BbLptC, BbLptA, BbLptB, BbLptF, BbLptG, and BbLptD, respectively.

### The N-terminal domain of BbLptD is periplasmic

**Figure 3.**
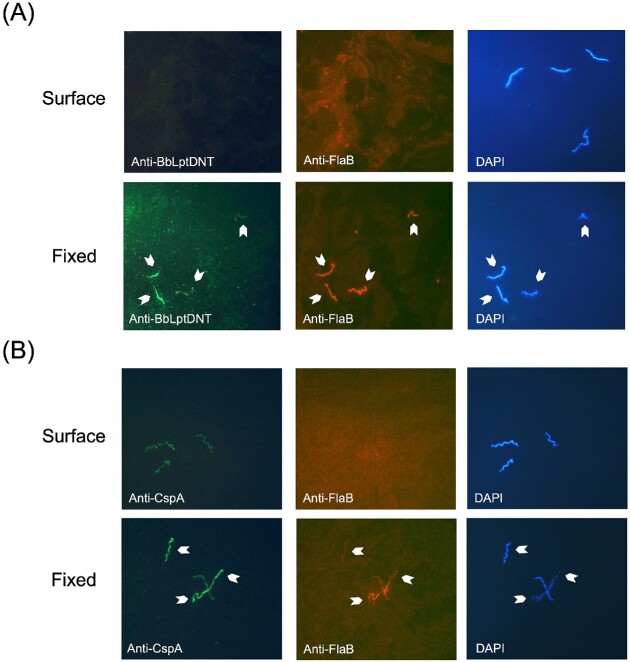
Immunofluorescence assays probing the N-terminal domain of BbLptD in *B. burgdorferi* strain B31 MI. (A) The localization of the N-terminal domain of BbLptD was examined using surface immunofluorescence assays and antibodies specific to the N-terminal domain of BbLptD (left-hand panels) or the periplasmic protein FlaB (middle panels). (B) As a control for surface fluorescence, cells were examined with antibodies specific to CspA, an abundant surface lipoprotein (left-hand panels) or the periplasmic protein FlaB (middle panels). In both (A) and (B), DAPI DNA staining was performed to visualize all the bacterial cells in the field of view (right-hand panels). Cells were incubated with the antibody cocktail either before (surface) or after permeabilization (fixed) of the *B. burgdorferi* OM to determine localization. White arrowheads indicate cells in each fixed-treatment panel.

The N-terminal beta-taco fold domain of *E. coli* LptD is known to be periplasmic and soluble when expressed independently (Chng et al. 2010, Freinkman et al. 2011, 2012). As described above and illustrated in Fig. 2, BbLptD is predicted to have a similar N-terminal beta-taco fold. To determine if the N-terminal domain of BbLptD was indeed periplasmic, immunofluorescence assays utilizing an antibody specific to the N-terminal domain of BbLptD (residues 29–291) were performed (Fig. 3). Specifically, the BbLptD N-terminal antibody was incubated either with intact *B. burgdorferi* cells or with *B. burgdorferi* cells that had been fixed through OM permeabilization with acetone (Fig. 3A). Given that antibodies cannot penetrate an intact OM, the lack of fluorescence on the borrelial surface indicates that the N-terminal domain of BbLptD is not localized on the surface of *B. burgdorferi* (Fig. 3A, upper left-hand panel). After OM permeabilization, incubation with the BbLptD N-terminal antibody results in fluorescence (Fig. 3A, lower left-hand panel). These results indicate that the N-terminal domain of BbLptD is not surface-exposed, consistent with our prediction that this domain is found in the periplasm. To ensure that *B. burgdorferi* cells were properly permeabilized in the fixed treatment and that the fragile OM remained intact in the surface treatment, all cells were also incubated with antibodies specific for the periplasmic protein FlaB (Fig. 3A, middle panels). All spirochetes in the microscopic field of view were also visualized through staining with the DNA-binding dye DAPI (Fig. 3A, right-hand panels). As an additional control to confirm proper detection of surface localized proteins by immunofluorescence, we performed the same set of experiments on *B. burgdorferi* cells that were incubated with antibodies specific for the abundant surface lipoprotein CspA (Brooks et al. 2005, Kenedy et al. 2009) (Fig. 3B). As expected, this resulted in fluorescence in both surface and fixed treatments (Fig. 3B, left-hand panels). Confirmation that the N-terminal domain of BbLptD is not surface localized is consistent with the computational structural modeling.

### BbLptA specifically interacts with the N-terminal domain of BbLptD

Previous studies in *E. coli* have demonstrated that the C-terminus of LptA specifically interacts with the N-terminal beta-taco domain of LptD (Freinkman et al. 2012). Since prior studies in our lab have identified BbLptD as an integral OMP (Kenedy et al. 2016), the finding that the N-terminal domain is not surface localized indicates it is likely localized to the periplasm. For these reasons, we chose to utilize this N-terminal domain rather than the entire BbLptD protein for these protein–protein interaction studies. To determine if the respective *B. burgdorferi* orthologs similarly interact, we utilized a copurification assay for the *B. burgdorferi* recombinant proteins. Specifically, the pACYCDuet-1 construct was utilized to coexpress BbLptA with an N-terminal His-tag and the N-terminal region of BbLptD (BbLptDNT) with a C-terminal S-tag in *E. coli*. This was done to ensure that the termini of each protein that were predicted to interact were free from any obstruction from the tags. For these experiments, supernatants were examined of the coexpressed proteins to illustrate the overall expression of the two proteins of interest. The copurification lanes display the His-tagged protein that was purified and whether it interacts with the coexpressed S-tagged protein of interest.

**Figure 4.**
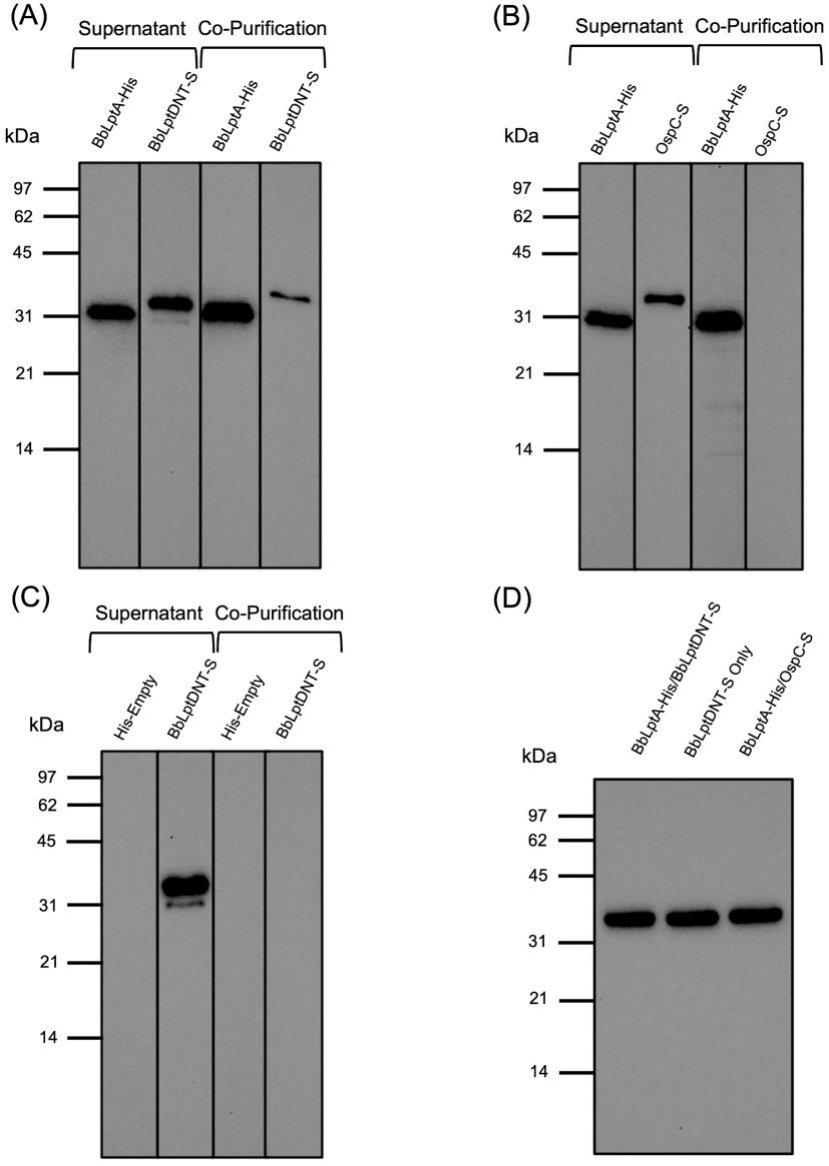
BbLptA and the N-terminal domain of BbLptD specifically interact. **(A)** Copurification assay of BbLptA-His and BbLptDNT-S. Supernatant from *E. coli* transformed with BbLptA-His/BbLptDNT-S pACYCDuet and copurifications were subjected to immunoblotting with His-tag specific antibody to detect BbLptA-His or S-tag specific antibody to detect BbLptDNT-S. **(B)** Copurification assay of BbLptA-His and OspC-S. Supernatant from *E. coli* transformed with BbLptA-His/OspC-S pACYCDuet and copurifications were subjected to immunoblotting with His-tag specific antibody to detect BbLptA-His or S-tag specific antibody to detect OspC-S. **(C)** Copurification assay of His-empty and BB0838NT-S. Supernatant from *E. coli* transformed with BbLptDNT-S pACYCDuet and copurifications were subjected to immunoblotting with His-tag specific antibody to ensure no expression of His-tagged proteins or S-tag specific antibody to detect BbLptDNT-S. **(D)** Anti-GAPDH immunoblot loading control for supernatants used in (A)**–**(C).

Using this system, we observed that BbLptDNT-S copurifies with BbLptA-His, indicating that BbLptA and the periplasmic region of BbLptD interact (Fig. 4A). Both of these proteins were well-expressed, as seen in the supernatant lanes of Fig. 4(A). To confirm that the interaction between BbLptDNT-S and BbLptA-His was specific, a copurification experiment was also performed with BbLptA-His and OspC-S utilizing coexpression of BbLptA-His and OspC-S in the pACYCDuet construct (Fig. 4B). OspC is an abundant *B. burgdorferi* protein, so this control ensures that there are no nonspecific interactions between BbLptA-His and other *B. burgdorferi* proteins or the S-tag itself. As seen in Fig. 4(B), OspC-S does not copurify with BbLptA-His. Additionally, BbLptDNT-S was expressed alone in pACYCDuet to ensure that the recombinant protein does not purify on the Ni-NTA resin in the absence of BbLptA-His. As shown in Fig. 4(C), BbLptDNT-S, despite being expressed at high levels in the supernatant, does not purify on the Ni-NTA resin alone. The combined controls in Fig. 4(B) and (C) indicate that the interaction between BbLptA-His and BbLptDNT-S is specific. A loading control using antibodies specific for the housekeeping protein GAPDH was also included (Fig. 4D) to ensure all of the supernatants were loaded equally.

### BbLptC specifically interacts with BbLptA

**Figure 5.**
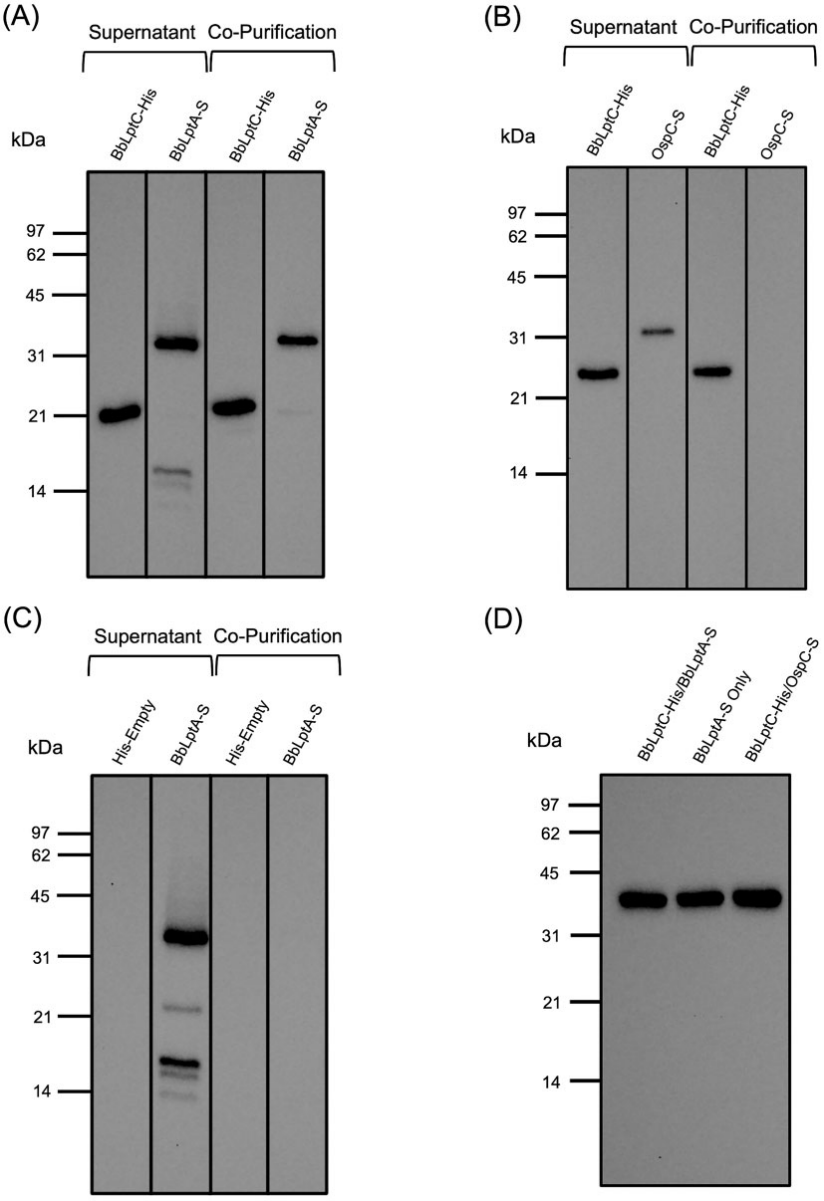
BbLptC and BbLptA specifically interact. **(A)** Copurification assay of BbLptC-His and BbLptA-S. Supernatant from *E. coli* transformed with BbLptC-His/BbLptA-S pACYCDuet and copurifications were subjected to immunoblotting with His-tag specific antibody to detect BbLptC-His or S-tag specific antibody to detect BbLptA-S. **(B)** Copurification assay of BbLptC-His and OspC-S. Supernatant from *E. coli* transformed with BbLptC-His/OspC-S pACYCDuet and copurifications were subjected to immunoblotting with His-tag specific antibody to detect BbLptC-His or S-tag specific antibody to detect OspC-S. **(C)** Copurification assay of His-empty and BbLptA-S. Supernatant from *E. coli* transformed with BbLptA-S pACYCDuet and copurifications were subjected to immunoblotting with His-tag specific antibody to ensure no expression of His-tagged proteins or S-tag specific antibody to detect BbLptA-S. **(D)** Anti-GAPDH immunoblot loading control for supernatants used in (A**)–**(C).

LptA and LptC have been found to interact as part of the LPT system in *E. coli* (Bowyer et al. 2011, Sperandeo et al. 2011, Schultz et al. 2013). Therefore, the pACYCDuet copurification system was also used to investigate the interaction between BbLptA and BbLptC. Similar to the experiments for BbLptA and BbLptDNT above, the pACYCDuet system was used to coexpress and copurify BbLptC-His and BbLptA-S in *E. coli* (Fig. 5A). BbLptC was expressed on the N-terminal His-tag to keep the C-terminus of the protein available for binding, as the C-terminus of LptC is necessary for interaction with LptA (Sperandeo et al. 2011). Both BbLptC-His and BbLptA-S were expressed in the supernatant (Fig. 5A). After purification of BbLptC-His, BbLptA-S was found to copurify with BbLptC-His (Fig. 5A), indicating an interaction between the two proteins. To confirm that the BbLptC-His and BbLptA-S interaction was not the result of nonspecific interactions, OspC-S was coexpressed with BbLptC-His in pACYCDuet and, separately, BbLptA-S was expressed alone in the pACYCDuet construct. As expected, there was no copurification observed in either control despite expression of each protein in the supernatant (Fig. 5B and C). The combined controls confirmed that the interaction between BbLptC-His and BbLptA-S is specific. The supernatants used in these experiments were normalized as illustrated by expression of GAPDH (Fig. 5D).

### Structural modeling of *B. burgdorferi* Lpt–ortholog interactions

**Figure 6.**
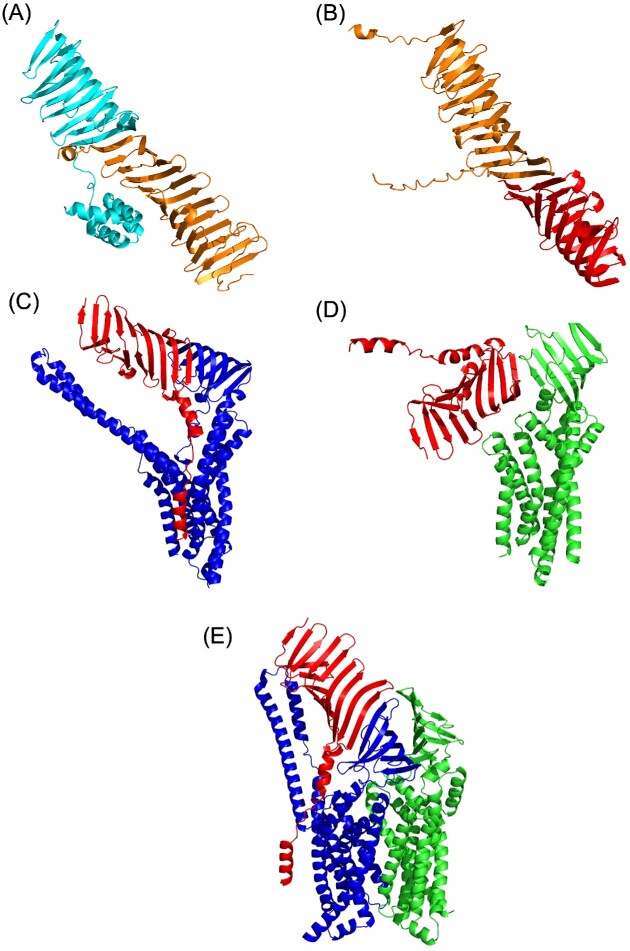
Multimer AlphaFold2.1 modeling of predicted interactions between *B. burgdorferi* Lpt proteins. **(A)** Multimer modeling and the predicted interaction interface between the N-terminal domain of BbLptD (cyan) and BbLptA (orange). The N-terminal domain of BbLptD corresponds to amino acids Q30-I271, and BB0465 was modeled without its signal peptide. **(B)** Multimer modeling and the predicted interaction interface between BbLptA (orange) and BbLptC (red). Both proteins were modeled without their signal peptides. **(C)** Multimer modeling and the predicted interaction interface between BbLptC (red) and BbLptF (blue). **(D)** Multimer modeling and the predicted interaction interface between BbLptC (red) and BbLptG (green). **(E)** Multimer modeling of the predicted interaction between BbLptC (red), BbLptF (blue), and BbLptG (green).

To further supplement the protein–protein interaction studies between BbLptA/BbLptDNT and BbLptC/BbLptA, each protein pair was subjected to AlphaFold2.1 multimer modeling (Fig. 6). BbLptA without the predicted signal peptide was submitted as a multimer with the N-terminal domain of BbLptD (Fig. 6A). These two proteins are predicted to interact at an approximate interface comprised of N-terminal residues R105–N118 of BbLptDNT and C-terminal residues Y209–Q221 of BbLptA to create a continuous beta-taco fold. AlphaFold2.1 provides a confidence score of this predicted interaction composed of the predicted template modeling (pTM) plus the interface predicted template modeling (ipTM). A weighted sum of these two numbers is used to provide an overall model confidence where a score closer to 1 indicates a more confident model and interaction interface (Evans et al. 2022). The confidence score for the interaction interface between BbLptA and BbLptDNT is 0.84 (Table 3), indicating that there is a high likelihood that this interface is where the interaction between BbLptA and BbLptDNT occurs. Additionally, the overall pLDDT of 88.22 of the two proteins indicates overall confidence in their structures (Table 3). Multimer modeling was also performed to examine the BbLptA and BbLptC interaction, both in the absence of their signal peptides. The model predicted an interaction at the C-terminal end of BbLptC and the N-terminal end of BbLptA (Fig. 6B). This interaction forms a cohesive beta-taco fold structure, similar as to the interaction between BbLptDNT and BbLptA shown in Fig. 6(A). The combined pLDDT of the BbLptC/BbLptA interaction was 87.62, indicating confidence in the overall predicted structure. Furthermore, the ipTM + pTM value was 0.84 (Table 3), which suggests high confidence in the predicted interaction interface at residues N162–N174 of BbLptC and residues F41–V52 of BbLptA.

**Table 3.** AlphaFold2.1 pLDDT scores and pITM + pTM of *B. burgdorferi* Lpt ortholog interactions.

Protein	pLDDT^†^	ipTM + pTM^‡^
BbLptA/BbLptDNT^§^	88.22	0.84
BbLptC/BbLptA	87.62	0.84
BbLptC/BbLptF	85.70	0.79
BbLptC/BbLptG	82.40	0.67
BbLptC/BbLptF/BbLptG	81.98	0.74

†: The pLDDT per residue score is averaged to give the numbers shown for each respective protein.

‡: The ipTM + pTM score is a measure of confidence in the predicted interaction. This is weighted 80% ipTM + 20% pTM.

§: BbLptDNT indicates the N-terminal domain (residues Q30–I271) was modeled in the multimer.

We also investigated the interaction of BbLptC and the putative inner membrane permease. Multimer modeling predicted a higher likelihood that the N-terminal region of the beta-taco fold of BbLptC interacts with BbLptF than BbLptG. Both models, however, do represent overall confident tertiary structures with pLDDTs of 85.70 and 82.40 for BbLptC/BbLptF and BbLptC/BbLptG, respectively (Table 3). Overall, the interaction model between BbLptC and BbLptF illustrates a much more cohesive structure, particularly in the connections of the beta-taco folds from each protein rather than that of the disjointed interaction shown between BbLptC and BbLptG (Fig. 6C and D, respectively). The predicted interaction interface was predicted to be between residues S34–V46 of BbLptC and residues Y224–Y234 of BbLptF. In addition to the structural models, the ipTM + pTM score of 0.79 for BbLptC/BbLptF indicates a much more likely interaction than the 0.67 score of the BbLptC/BbLptG multimer (Table 3). This observation also is consistent with previous studies suggesting that LptC interacts specifically with LptF rather than LptG in *E. coli* (Benedet et al. 2016). To support this finding, BbLptC, BbLptF, and BbLptG were modeled together as a trimer using AlphaFold2.1, and the result was consistent with BbLptC interacting preferentially with BbLptF (Fig. 6E). Additionally, the overall pLDDT was considered confident with a score of 81.98, and the predicted interface of the interaction was close to the BbLptC/BbLptF ipTM + pTM at 0.74 (Table 3).

### Generation of an IPTG regulatable BbLptD mutant

**Figure 7.**
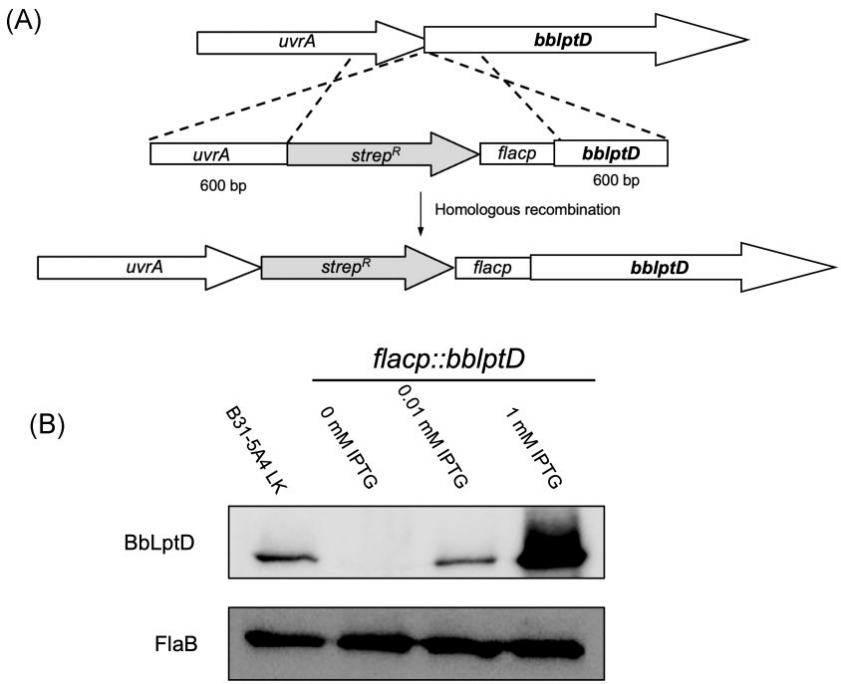
Generation of an IPTG-regulatable BbLptD mutant in the virulent *B. burgdorferi* strain B31-5A4 LK. **(A)** The *flacp* IPTG-regulatable promoter and the streptomycin resistance cassette were inserted upstream of *bblptD* through homologous recombination. Homologous recombination was achieved by first generating a construct in which the regions 600 bp upstream and downstream of the insertion site were cloned into pBluescript SK +. The streptomycin cassette as well as the *flacp* promoter were then cloned into the construct. The final construct was electroporated into *B. burgdorferi* B31-5A4 LK, and a streptomycin resistant clone was selected and designated *flacp*::*bblptD*. A full plasmid analysis was also performed on the mutant strain (data not shown). **(B)** IPTG dose-dependent expression of BbLptD in the *flacp::bblptD* mutant strain. The *B. burgdorferi flacp::bblptD* mutant strain was cultivated in 0, 0.01, or 1.0 mM IPTG, and whole-cell lysates of the mutant and wildtype strains were subjected to immunoblot with BbLptD antibodies. Whole-cell lysates immunoblotted with FlaB antibodies are also shown as controls for equal loading.

Previous genome-wide transposon mutagenesis studies in *B. burgdorferi* yielded no mutants with transposons in *bb0838* (referred to as *bblptD* forward) (Lin et al. 2012). This suggests that *bblptD* and the protein it encodes are essential in *B. burgdorferi*, which is consistent with observations that LptD is essential in Gram-negative organisms (Braun and Silhavy 2002, Werneburg et al. 2012). Given these prior observations, it was not surprising that we were unable to obtain a *bblptD* deletion mutant in *B. burgdorferi* after repeated attempts. Therefore, we generated an IPTG-regulatable *bblptD* mutant to utilize in further studies. As illustrated in Fig. 7(A), the IPTG-inducible *flacp* promoter was inserted upstream of *bblptD*. We confirmed all plasmids found in the parental B31-5A4 LK strain were also present in the mutant strain, designated *flacp::bblptD*. Analysis of whole cell lysates of uninduced (0 mM IPTG) *flacp::bblptD* vs. B31-5A4 LK wild-type organisms illustrates a greatly reduced level of BbLptD expression (Fig. 7B). Expression of BbLptD is restored almost back to wild-type levels with only 0.01 mM IPTG added to the growth media and is overexpressed with 1 mM IPTG added to the media (Fig. 7B). As expected, FlaB levels remained consistent regardless of the amount of supplemented IPTG added to the growth media (Fig. 7B).

### Lipoprotein CspA is transported to the surface despite BbLptD down-regulation

While it is known that *B. burgdorferi* encodes numerous surface-exposed lipoproteins (Fraser et al. 1997, Brightbill et al. 1999), the process involved in surface localization of these lipoproteins has long remained a question. It has previously been proposed that all surface-exposed lipoproteins in *B. burgdorferi* are transported to the surface through the same general “ flippase” (Zückert 2014). Because lipoproteins have a similar amphiphilic nature to LPS and serve as a component of OM asymmetry in *B. burgdorferi*, we sought to investigate these lipoproteins as the potential cargo of the LPT-orthologous system in *B. burgdorferi*. We utilized the IPTG regulatable BbLptD mutant to determine if lipoproteins can be localized to the surface of *B. burgdorferi* when BbLptD is down-regulated. To examine this possibility, we performed surface proteolysis assays combined with immunofluorescence on whole cells of B31-5A4 LK wild-type organisms or on *flacp::bblptD* cultures supplemented with either 0, 0.01, or 1 mM IPTG.

**Figure 8.**
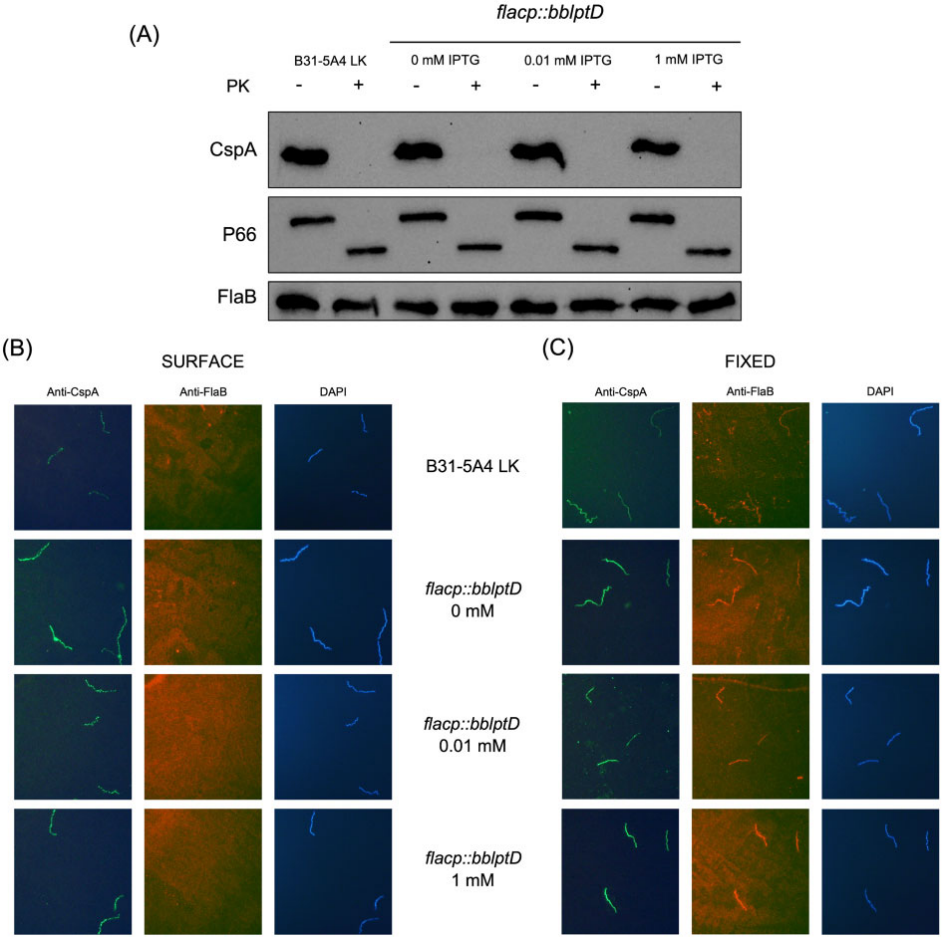
Borrelial lipoproteins are transported to the surface despite BbLptD down-regulation. **(A)** Proteinase K (PK) surface proteolysis of the abundant *B. burgdorferi* surface lipoprotein CspA. PK assays were performed on the B31-5A4 LK wild-type and the *flacp::bblptD* mutant grown with either 0 mM IPTG, 0.01 mM IPTG, or 1 mM IPTG. −/+ indicates the absence or presence of PK, respectively. After PK incubation, whole-cell lysates were subjected to immunoblot with CspA specific antibodies. The periplasmic FlaB protein and the known OMP P66 were included as controls. **(B)** Surface immunofluorescence assays of surface lipoprotein CspA performed on B31-5A4 LK wild-type, *flacp::bblptD* 0 mM IPTG, *flacp::bblptD* 0.01 mM IPTG, or *flacp::bblptD* 1 mM IPTG. Cells were incubated with CspA and FlaB antibodies prior to permeabilization of the OM. **(C)** Immunofluorescence assays of surface lipoprotein CspA performed on B31-5A4 LK wild-type, *flacp::bblptD* 0 mM IPTG, *flacp::bblptD* 0.01 mM IPTG, or *flacp::bblptD* 1 mM IPTG. Cells were incubated with CspA and FlaB antibodies after permeabilization of the OM. FlaB antibodies were included in immunofluorescence assays to ensure the OM remained intact during the surface assays, and DAPI counter-staining was included to identify all organisms in a given field in (B) and (C).

For surface proteolysis assays, organisms were incubated either with or without addition of proteinase K (PK) (Fig. 8A). Since PK cannot penetrate the OM, only surface-exposed proteins are degraded when cells are treated with PK. Following surface proteolysis, each PK-treated sample and the corresponding control was immunoblotted with antibodies specific for CspA, an abundant surface-exposed lipoprotein in *B. burgdorferi* (Cordes et al. 2004). As shown in Fig. 8(A), when B31-5A4 LK wild-type cells are treated with PK, there is full degradation of CspA, indicating it is surface exposed as expected. Similarly, when the BbLptD regulatable strain was supplemented with 0.01 or 1 mM IPTG, we found that CspA is fully degraded and, therefore, surface-exposed (Fig. 8A). Interestingly, we also observed that CspA was fully degraded from the borrelial surface when the BbLptD regulatable strain was cultured without IPTG (Fig. 8A). Full degradation of CspA when BbLptD is not induced for expression indicates CspA is not dependent on BbLptD for transport to the *B. burgdorferi* surface. As a control, we also examined PK-treated and untreated cells by immunoblot with P66-specific antibodies, a known membrane-spanning, surface exposed OMP. P66 was degraded and resulted in a smaller, 50 kDa band as shown previously (Curtis et al. 2022). Lastly, the periplasmic FlaB protein was also used for immunoblots on the same whole-cell lysates to confirm the organisms’ OMs were not disrupted and that all degradation was indicative of only surface protein degradation (Fig. 8A).

To more closely examine whether surface-exposure of CspA is dependent on BbLptD expression, we also performed surface localization immunofluorescence assays on B31-5A4 LK wild-type cells and the regulatable mutant grown in 0, 0.01, or 1 mM IPTG (Fig. 8B). Cells were incubated with CspA antibodies either before or after permeabilization of the OM. Since antibodies do not penetrate the OM of *B. burgdorferi*, immunofluorescence observed on intact cells indicates surface localization. As shown in Fig. 8(B), wild-type and all mutant organisms strongly fluoresced with the CspA antibody, indicating that CspA is surface exposed regardless of BbLptD expression (Fig. 8B, left-hand panels). To ensure that OMs were not disrupted prior to antibody incubation, cells were also incubated with FlaB-specific antibodies. In the unfixed, intact cells (Fig. 8B, center panels), there is no fluorescence of the FlaB antibodies, indicating the OMs were intact. As a control, the same experiment was performed on fixed cells and assayed with CspA (Fig. 8C, left-hand panels) or FlaB (Fig. 8C, center panels) antibodies and, as expected, both CspA and FlaB were observed by fluorescence. To visualize all spirochetes in each microscopic field, all slides were stained with DAPI (Fig. 8B and C, right-hand panels). Taken together, the surface proteolysis and immunofluorescence assays illustrate that the down-regulation of BbLptD in the *flacp::bblptD* 0 mM IPTG mutant does not affect the surface localization of CspA, and strongly suggests that BbLptD and the *B. burgdorferi* LPT system are not required for lipoprotein transport to the surface.

### Structural modeling of the *B. burgdorferi* BbLptA with borrelial glycolipids

Since the data indicated surface lipoproteins do not require BbLptD to be transported to the surface, we next investigated the possibility that the amphiphilic glycolipids could be the cargo. To begin these studies, we used computational structural modeling. We focused these studies on interactions between BbLptA and the two major *B. burgdorferi* glycolipids, BbGL-I and BbGL-II (Figure S1, Supporting Information). Using AlphaFold2.1, BbLptA was modeled similarly to the solved structures of the LptA proteins of Gram-negative organisms. Specifically, it has a tightly packed interior composed entirely of hydrophobic residues and a surface highly enriched in hydrophilic and polar residues. Since the previously solved structures of LptA proteins have been done without bound cargo, we sought to model BbGL-I and BbGL-II in the hydrophobic interior of BbLptA to explore the potential interaction between glycolipids and BbLptA. The beta-taco of BbLptA was closed, which excludes traditional docking approaches from placing the glycolipids in the hydrophobic interior, so we used a combination of tools to explore the flexibility of BbLptA with BbGL-I and BbGL-II.

First, a SILCS (Site Identification by Ligand Competitive Saturation) simulation (Guvench and MacKerell 2009) was performed. This utilizes combined grand canonical Monte Carlo (GCMC) with molecular dynamics (MD) in a mixed-solvent environment containing small probes of a variety of biochemical characteristics such as propane, benzene, methanol, acetate, and methylammonium, among others (Lakkaraju et al. 2015). This mix of small molecules can aid in the identification of binding regions of larger molecules based on shared characteristics between the small molecules and large molecules (MacKerell et al. 2020). Regions with higher probe residency time during the simulations suggest favorable interactions, and interaction free energy maps of various functional groups, FragMaps, can be generated based on these probe residency times (Guvench and MacKerell 2009). Because of the flexibility that MD affords, SILCS has been used to identify occluded or cryptic pockets not present in experimental or Alphafold apo protein structures (Lakkaraju et al. 2015).

Using the FragMas from the SILCS simulations, we combined SILCS–Hotspots and shape overlay tools to place the glycolipids within the interior pocket of the structural model of BbLptA. SILCS–Hotspots performs Monte Carlo-based “docking” of fragments into the FragMaps across the entire protein to identify binding sites for each fragment (MacKerell et al. 2020). Because of its large size, BbGL-I was decomposed into smaller fragments, and binding conformations for each fragment were predicted at each interaction site. To place the entire glycolipid into BbLptA, we sought to find a low-energy conformation of BbGL-I that also resembled the same favorable interactions in the interior of the beta-taco that were curated from the SILCS-Hotspots results. A set of over 20 000 low energy conformations was generated by OMEGA, and ROCS (Rapid Overlay of Chemical Structures) was used to identify conformations of BbGL-I that overlaid with the hotspot BbGL-I fragments ([Bibr bib84][Bibr bib84], OpenEye Scientific Software 2010, Hawkins et al. 2007, 2010). The pose with the highest overlay score that did not protrude through the beta sheets was selected.

Finally, because the closed model of BbLptA clashed with the glycolipid pose, we employed a MD simulation to allow the protein to undergo a conformational change to accommodate the glycolipid. The complex was solvated, neutralized, minimized, and then equilibrated in the presence of restraints for 100 ps. The restraints were then removed and a 200 ns MD simulation was performed using GROMACS (Groningen Machine for Chemical Simulations) (Abraham et al. 2015). BbGL-II was modeled into the structure of BbLptA in final frame of the MD simulation of the BbGL-I complex using the same protocol, except the final BbGL-I conformation was used as the query for ROCS.

**Figure 9.**
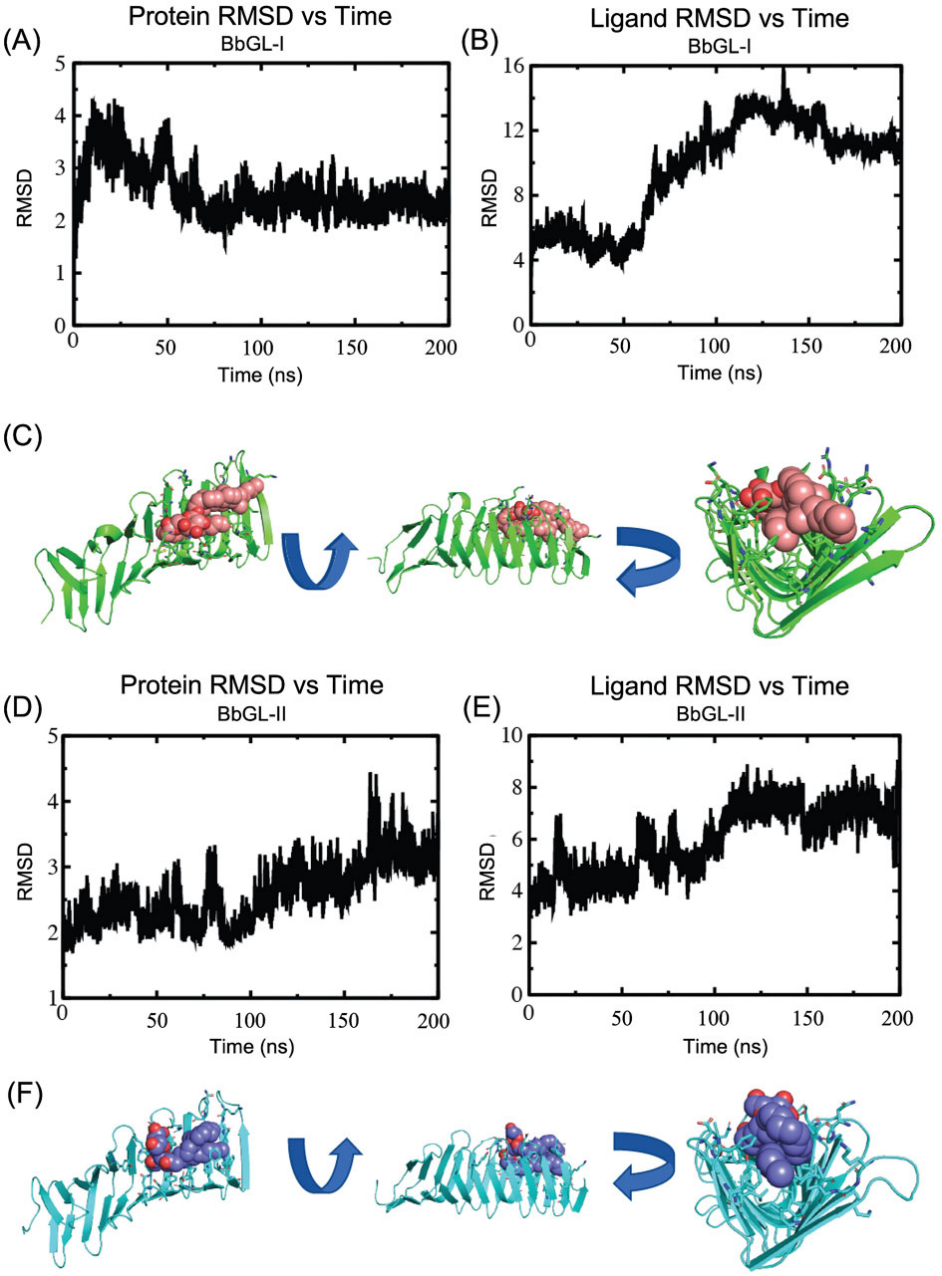
MD simulations converged to a final model of BbLptA with two glycolipids. **(A)** Plot of C-alpha RMSD vs. time of BbLptA in the simulation with BbGL-I relative to its position following equilibration. Upon release of the restraints, there is a rapid 4 Å change in RMSD, while after 100 ns, it converges to around 2.5 Å from the starting structure. **(B)** Plot of ligand RMSD vs. time of BbGL-I relative to its position following equilibration. Upon release of the restraints, there is a rapid 4 Å change in RMSD, while after 50 ns, BbGL-I converges to around 12 Å from the starting structure. This corresponds to the acyl chain folding under the carbohydrate and sterol. **(C)** The beta taco fold of BbLptA (green) with BbGL-I (salmon) following 200 ns MD simulation. The beta taco has opened to allow the sterol group and acyl chain to insert into the hydrophobic interior, while the carbohydrate is oriented outwards to interact with the hydrophilic surface of BbLptA or solvent. The red atoms on BbGL-I indicate oxygens present in the sugar group or glycerol. The first arrow represents a 90° rotation along the *x*-axis. The second arrow represents an additional 90° rotation along the *y*-axis. **(D)** Plot of C-alpha RMSD vs. time of BbLptA in the simulation with BbGL-II relative to its position following equilibration. Upon release of the restraints, there is a rapid 2 Å change in RMSD, while after 170 ns, it converges to around 3 Å from the starting structure. **(E)** Plot of ligand RMSD vs. time of BbGL-II relative to its position following equilibration. Upon release of the restraints, there is a rapid 4 Å change in RMSD, while after 100 ns, it converges to around 7 Å from the starting structure. This corresponds to the carbohydrate orienting itself in the direction of the OM. **(F)** The taco fold of BbLptA (cyan) with BbGL-II (purple) following 200 ns MD simulation. As with BbGL-I, the beta-taco has opened to allow the acyl chains to insert into the hydrophobic interior, while the carbohydrate is oriented outwards to interact with the hydrophilic surface of BbLptA or solvent. The red atoms on BbGL-I indicate oxygens present in the sugar group or glycerol. The first arrow represents a 90° rotation along the *x*-axis. The second arrow represents an additional 90° rotation along the *y*-axis.

During the MD simulations, both the protein and ligand had a rapid shift away from the starting structure upon the release of the restraints as the protein and ligand both moved into lower energy conformations; however, in each simulation the protein and ligand seemed to converge toward a lower-energy conformation at around 100 ns (Fig. 9A/B and D/E). The final frame of each trajectory provides a snapshot of how each glycolipid potentially interacts with the protein as it slides through BbLptA (Fig. 9C/F). Given that the glycolipids are adjacent to where BbLptC would be located, these models represent how the glycolipid may bind after they are passed from BbLptC to BbLptA. In both models, the sugar is oriented away from where BbLptC would be located and would theoretically travel toward the OM. The sugar of the glycolipid is pointed upward to interact with the hydrophilic surface residues and solvent, while the lipid tails are oriented toward the inner membrane (Fig. 9C/F). Further empirical studies will be needed to better define this mechanism.

## Discussion

Previously, BB0807, BB0808, BB0465, BB0466, and BB0838 have been identified as Lpt-related proteins in *B. burgdorferi* (Putker et al. 2015, Kenedy et al. 2016). We further examined the similarity between the borrelial proteins and known Lpt orthologs using a combination of genetic and structural data. Given that the genetic locus encoding *lptC* in Gram-negative organisms is always found to consist of *lptC* directly upstream of *lptA* and *lptB*, we hypothesized that *bb0464* was the *lptC* ortholog in *B. burgdorferi*. Subsequent structural modeling confirmed BB0464 as the LptC ortholog as the structure was remarkably similar to other LptC proteins. Additionally, LptC has been shown to preferentially interact with LptF in *E. coli* (Benedet et al. 2016). The multimer modeling performed here predicted that BB0464 preferentially interacts with BB0807. Combined with the better RMSD scores for BB0807 aligned to LptF and BB0808 aligned to LptG, respectively, we have further characterized BB0807 as the LptF ortholog and the BB0808 as the LptG ortholog. The genetic and structural analyses also allowed us to confirm BB0465, BB0466, and BB0838 as LptA, LptB, and LptD orthologs, respectively. Accordingly, the *B. burgdorferi* proteins BB0464, BB0465, BB0466, BB0807, BB0808, and BB0838 should be referred to, respectively, as BbLptC, BbLptA, BbLptB, BbLptF, BbLptG, and BbLptD moving forward.

Interestingly, the structural models of BbLptD and BbLptC differed slightly from their Gram-negative counterparts. Specifically, these differences included the alpha helices at the N-terminal domain of BbLptD and the prediction that BbLptC is a lipoprotein. Differences in overall protein structure are not surprising considering the function of the *B. burgdorferi* LPT system clearly diverges from LPS transport. Additionally, while Fig. 2 illustrates a single BbLptA molecule linking BbLptC to BbLptD, it is important to note that LptA oligomerizes to span the length of the periplasm in other organisms (Santambrogio et al. 2013). The periplasmic space of *B. burgdorferi* is ∼160 Å (Charon et al. 2009), and the respective lengths of each beta-taco fold (measured through PyMOLv2.4.0; Schrödinger 2020) of BbLptC, BbLptA, and the N-terminal domain of BbLptD are 41.9, 55.2, and 52.9 Å. The combined approximation of 150 Å indicates that it is entirely possible that BbLptA functions as a monomer in this system. However, given the substantially larger periplasmic space present where the endoflagella are located (Charon et al. 2009), it is also possible that multiple BbLptA proteins are needed in these regions. Further studies would be needed to resolve these questions.

While the genetic and structural data strongly indicated *B. burgdorferi* encoded a novel LPT transport system, we sought to determine empirically that these orthologs do indeed interact, which would be required for them to form a bridge between the inner and OMs. We examined the interactions between the N-terminal periplasmic domain of BbLptD with BbLptA as well as BbLptC with BbLptA. These three components of the LPT system are known to be the periplasmic bridge proteins in other organisms (Laguri et al. 2017), and we wanted to confirm the borrelial orthologs also interacted similarly, which would indicate they could link the inner and OMs in *B. burgdorferi*. Utilizing the pACYCDuet coexpression and copurification system, we observed that BbLptC interacts with BbLptA and that BbLptA interacts with the periplasmic domain of the OMP BbLptD. The combined findings are most consistent with the BbLptC/BbLptA/BbLptD proteins interacting and providing the periplasmic bridge for the LPT system in *B. burgdorferi*. Future studies examining the sites of interaction and the residues involved in the interactions between these proteins will be necessary to fully understand which residues are most relevant to these protein–protein interactions.

*B. burgdorferi* contains six of the seven orthologs to Gram-negative Lpt proteins, but it lacks an LptE ortholog. LptE is essential in Gammaproteobacteria and is known to form a stable complex with LptD with LptE residing inside the beta-barrel of LptD (Chng et al. 2010, Botos et al. 2016). LptE has been shown to play several roles in the LPT system including, but not limited to, acting as a “ plug” to the beta-barrel in LptD to inhibit continuous LPS transport (Grabowicz et al. 2013), binding to and inhibiting aggregation of LPS to allow proper transport (Malojčić et al. 2014), and being essential to the folding of LptD through stabilization and proper oxidation of the two di-sulfide bonds in LptD (Ruiz et al. 2010, Chimalakonda et al. 2011, Lo Sciuto et al. 2018). LptE is known to be the least conserved protein in the LPT system, and previous studies have found difficulties in identifying LptE orthologs in other organisms outside of the Gammaproteobacteria group (Putker et al. 2015). Given the general lack of sequence conservation seen in the *B. burgdorferi* LPT-system orthologs, this is one possible explanation for the lack of identification of an LptE ortholog in *B. burgdorferi*. However, it is also possible that there is no LptE ortholog in *B. burgdorferi* because it is not essential to the function of the LPT-orthologous system. BbLptD does not contain cysteines, so one of the known roles of LptE in assisting di-sulfide bond formation would not be a required function in *B. burgdorferi*. Additionally, it may also be possible that an LptE ortholog is not essential for the transport of the yet-to-be-identified cargo of this system, as the other roles of LptE all involve direct interaction with LPS.

With the presence of an inner membrane permease BbLptF/BbLptG and the ATP-binding cassette, consisting of a homodimer of BbLptB, it is very likely that the novel LPT system in *B. burgdorferi* transports an OM constituent to the surface of this spirochete. What the cargo for the borrelial LPT system might be is still unclear. Possible candidates include two major OM localized constituents that contain fatty acids and have a similar amphiphilic nature to LPS. These are the surface lipoproteins and the unique borrelial glycolipids. While both the lipoproteins and the glycolipids of *B. burgdorferi* are found to be immunogenic and contribute to disease pathogenesis (Schroder et al. 2008, Stübs et al. 2009, 2011, Kenedy et al. 2012), it is unknown how these major OM constituents are transported to the surface of this spirochete. Given that the *B. burgdorferi* genome does not likely encode specific transporters for each of the more than 100 known surface lipoproteins (Schulze and Zuckert 2006, Zückert 2014), it has been proposed that there is a general lipoprotein flippase that can transport lipoproteins from the periplasm to the surface (Zückert et al. 2004). It is tempting to speculate that this LPT-orthologous system in *B. burgdorferi* may serve as a route for lipoprotein surface localization, particularly due to the amphiphilic nature of the lipoproteins and the membrane asymmetry they provide in *B. burgdorferi*. We investigated this possibility using the regulatable BbLptD mutant grown in 0 mM IPTG, which results in down-regulation of BbLptD expression at a level where it is not even observed by immunoblot analysis. Even with the lack of observable BbLptD, we observed no change in the surface localization of the major surface lipoprotein CspA. The observation that CspA is still localized to the surface of *B. burgdorferi*, even when BbLptD is not expressed or, at a minimum, down-regulated to an extremely low level, suggests that BbLptD and the LPT system do not function as a lipoprotein transport system in *B. burgdorferi*.

A recent report has emerged from He et al. (2023) addressing similar questions proposed here. The investigators had similar computational findings about the presence of a *B. burgdorferi* LPT system, and we have provided further empirical evidence that this system exists in this spirochete and that the proteins within the system interact. The conclusions from their study, however, are different from our observations. He et al. (2023) determined that surface lipoproteins, including CspA, are not properly localized to the surface when BbLptD is down-regulated, while we found that CspA is surface localized independent of BbLptD expression. It should be noted, however, that the methodology to create a BbLptD knockdown system between the two studies differed significantly. Here, we use a well-established method (Caimano et al. 2007, Gilbert et al. 2007, Lenhart and Akins 2010, Dunn et al. 2015, Iqbal et al. 2016, Drecktrah and Samuels 2018) to regulate the expression of BbLptD through an inducible promoter. He et al. (2023) used a newer CRISPR interference *B. burgdorferi* system to knockdown BbLptD (Murphy et al. 2023). Overall, our data led us to conclude that, rather than lipoproteins, the glycolipids are the likely cargo of the *B. burgdorferi* LPT system—a possibility that He et al. (2023) do not presume, but also do not exclude.

The conjecture that glycolipids are the most likely cargo of the *B. burgdorferi* LPT system is consistent with the fact that *Treponema pallidum*, another spirochete that lacks LPS, also contains a predicted LPT system (Putker et al. 2015, Hawley et al. 2021). Unlike *B. burgdorferi*, however, *T. pallidum* does not have an abundance of surface-exposed lipoproteins and, in fact, only contains three potential surface-exposed lipoproteins (Radolf and Kumar 2018). *T. pallidum* is similar to *B. burgdorferi* in that it also contains specific glycolipids (Radolf et al. 1995). Therefore, it seems much more likely that the role of the LPT system in these spirochetes would be to transport glycolipids. Considering the dearth of surface-exposed lipoproteins in *T. pallidum*, we would also propose that glycolipids are the most likely cargo of the treponemal LPT system, which was also previously proposed by Hawley et al. (2021). Interestingly, other LPS-lacking diderms such as *Thermus thermophilus* and *Thermotoga maritima* also contain Lpt-orthologs (Putker et al. 2015) and possess their own unique membrane glycolipids (Manca et al. 1992, Leone et al. 2006) but do not encode surface lipoproteins. This is again consistent with our suggestion that glycolipids are the cargo for these LPT systems in organisms that lack LPS.

The BbLptA/BbGL-I and BbLptA/BbGL-II computational modeling provides insight into the possibility that the borrelial glycolipids are indeed the cargo of the LPT system in *B. burgdorferi*. This was illustrated based on the identification of putative binding sites for glycolipids inside the beta-taco fold using the SILCS technology. Subsequent docking model MD simulations predicted a 3D model of the binding of the glycolipids to the proteins. The buried lipids make nonspecific hydrophobic interactions in the interior of the beta-taco while the carbohydrate interacts with solvent and the hydrophilic surface side chains. The predicted binding poses are consistent with the ATPase, BbLptB, providing the energy to slide the glycolipid through the channel by pushing another glycolipid behind it. Based on the favorable nature of the glycolipid–protein interaction, the models propose a reasonable method of transport of glycolipids through BbLptA. Future studies utilizing regulatable mutants of the *B. burgdorferi* Lpt-orthologs will be necessary to definitively determine if glycolipids are transported by this system.

With so few trans-envelope transport/export systems having been identified in *B. burgdorferi*, much is still unknown about this spirochete’s OM biogenesis. The identification of a novel LPT system will have high impact on future *B. burgdorferi* OM biogenesis studies and may also provide a novel target for antimicrobials. While doxycycline and other broad spectrum antibiotics are very effective in treating Lyme disease, the USA has seen a large increase in Lyme disease without any indication of a future decline in cases (Stone et al. 2017). This has led to discussion of control and/or elimination of *B. burgdorferi* in the tick reservoir (Dolan et al. 2011, Richer et al. 2011, Bernard et al. 2020). Doxycycline containing bait targeting mice in Lyme disease endemic regions have proved effective in drastically reducing the number of small mammals and ticks infected with *B. burgdorferi*, but on a large scale, this strategy causes concern on the development of antibiotic resistance to the most effective Lyme disease drug (Dolan et al. 2011, Bernard et al. 2020). The identification of this novel and essential transport system in *B. burgdorferi* could provide an additional target to combat this issue. Additionally, the identification of an LPT-orthologous system in LPS-lacking *B. burgdorferi* provides novel insight into the function of the LPT system in general. Perhaps, the sole and specific role of this system is not to transport LPS, but rather to transport a variety of fatty acid-containing molecules to the surface of different diderm species. We have provided computational and empirical evidence of the presence of such a system in *B. burgdorferi*. We have identified an LPT-orthologous system in this spirochete and have thus far provided evidence that lipoproteins are not transported by this novel LPT system in *B. burgdorferi*. While we hypothesize here that the borrelial glycolipids are the likely cargo of the LPT-orthologous system in *B. burgdorferi*, further mechanistic studies will be required to examine this issue for confirmation.

## Supplementary Material

ftad014_Supplemental_Figure
