# Supplementary figures and images for: Identification of a novel transport system in Borrelia burgdorferi that links the inner and outer membranes
Source: Pathog Dis. 2023 Jun 29;81:ftad014. doi: 10.1093/femspd/ftad014 (PMC10353723; doi:10.1093/femspd/ftad014)

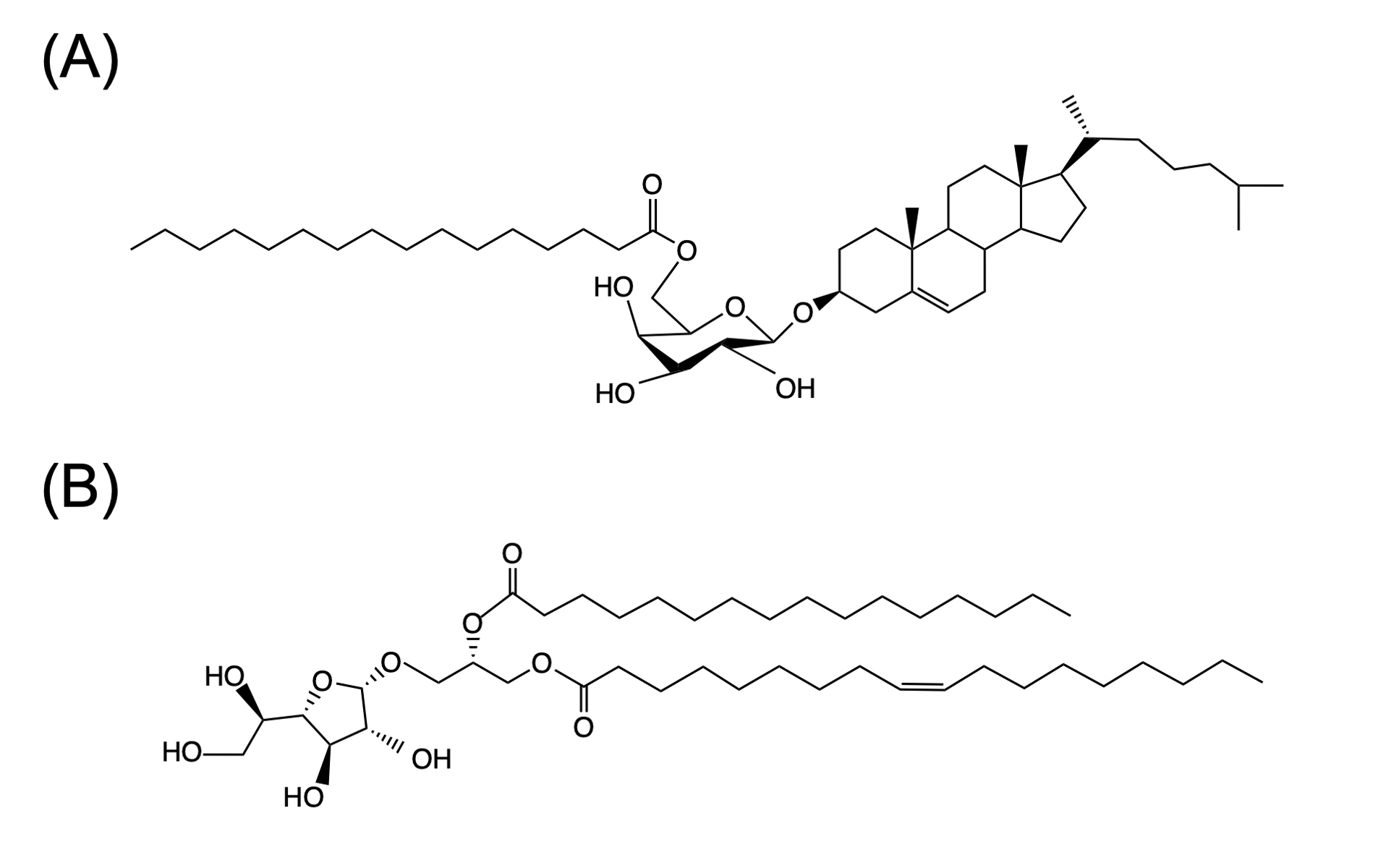

Supplement: ftad014_Supplemental_Figure [file ftad014_supplemental_figure.zip › Lipid Supplemental Fig High Quality.jpg]
